# A clear approach: Hemostatic gel as a novel adjunct for pediatric upper gastrointestinal bleeding

**DOI:** 10.1002/jpr3.70000

**Published:** 2025-02-03

**Authors:** Natalia Plott, Audra Rougraff, Paroma Bose, Kyla M. Tolliver, Shamaila Waseem, Brett J. Hoskins

**Affiliations:** ^1^ Department of Pediatrics, Division of Pediatric Gastroenterology, Hepatology, and Nutrition Indiana University School of Medicine, Riley Hospital for Children at IU Health Indianapolis Indiana USA

**Keywords:** hemostasis, pediatrics, purastat, self‐assembling peptide RADA16, therapeutic endoscopy

## Abstract

Pediatric upper gastrointestinal bleeding (UGIB) is a significant clinical concern, with a mortality rate of approximately 2%. Endoscopic management of UGIB in children includes various techniques such as injections, mechanical devices, thermal therapies, and topical agents. PuraStat®, a clear hemostatic gel, has been used in adults to create a physical barrier for hemostasis without obscuring the endoscopic view. However, its use in pediatric UGIB has not been well‐documented. A review of four pediatric cases where PuraStat® was used to treat UGIB showed that it was applied as an adjunct to other hemostatic methods like clip placement or epinephrine injections, and in one case, as monotherapy for a large duodenal ulcer/site of recently contained perforation. The gel was easy to use, appeared to be beneficial, and was well‐tolerated in this small cohort, although conclusions regarding its safety and efficacy are limited by the sample size.

## Abstract (highlights)

**What is Known**

- Pediatric upper gastrointestinal bleeding (UGIB) has a mortality rate of ~2%.
- Endoscopic treatments for UGIB in children include injections, mechanical devices, thermal therapies, and topical agents.
- Hemostatic powders are commonly used but may obscure visibility and clog the catheter.
- PuraStat®, a transparent hemostatic gel used successfully in adult UGIB, lacks sufficient data on its safety and efficacy in pediatric patients.

**What is New**

- PuraStat® was easy to use, appeared to be beneficial, and was well‐tolerated as adjunctive hemostatic therapy in four pediatric UGIB cases.
- PuraStat's® transparent gel preserved endoscopic visibility, addressing potential limitations seen with hemostatic powders.
- This case series suggests the potential for PuraStat® as a promising adjunctive hemostatic option for pediatric UGIB.

**Abbreviations**
EGD: esophagogastroduodenoscopy
UBIG: upper gastrointestinal bleeding
ΔHb: change in hemoglobin

## 1 INTRODUCTION

Upper gastrointestinal bleeding (UGIB), defined as bleeding from a site proximal to the ligament of Treitz, accounts for 20% of all gastrointestinal bleeding in children with a reported incidence of 6.4%.^1^, ^2^ The causes and prevalence of UGIB vary by age and severity of blood loss. While some UGIB cases require minimal intervention, severe and life‐threatening cases may need additional measures. Approximately, 0.5% of pediatric hospitalizations and 0.4% of critical care hospitalizations are due to UGIB, with prolapse gastropathy syndrome (12.7%), gastric erosions/ulcers (10.8%), erosive esophagitis (9.5%), and duodenal erosions/ulcers (8.2%) as the most common causes of nonvariceal bleeding in children in North America and Europe.^1^, ^2^, ^3^ Pediatric UGIB has a reported mortality rate of about 2%, with endoscopic therapeutic intervention being a protective factor.^4^ Treatment options include injections, mechanical devices, thermal therapies, and topical agents. Hemostatic powders have recently become common topical agents used in pediatrics, though may be difficult to use at times due to catheter clogging or obscured visibility after powder deployment.^5^ PuraStat® (3‐D Matrix, Inc.), a biocompatible synthetic viscous gel, is a novel hemostatic agent used in adults for hemostasis. Application is achieved by delivering a controlled volume of the gel to adequately coat the area of interest using a dedicated 220 cm catheter provided with the product. The catheter can be introduced through the working channel of any diagnostic or therapeutic endoscope with a channel diameter of 2.8 mm or larger. Through the process of RADA16 self‐assembling peptide technology, the hydrogel matrix forms a transparent physical barrier, resulting in hemostasis while maintaining visibility. Several adult studies have reported successful initial hemostasis in 88%–91% of cases, though pediatric data are lacking.^6^, ^7^ PuraStat® has also been utilized for bleeding prophylaxis following endoscopic mucosal resection and endoscopic submucosal dissection in adult studies.^8^, ^9^ In this case series, we report four pediatric UGIB cases where PuraStat® was used as either adjunctive therapy or monotherapy for ulcers to assess its feasibility and application in children.

## 2 METHODS

This retrospective single‐center case series included four pediatric patients who underwent esophagogastroduodenoscopy (EGD) for UGIB managed using standard endoscopic techniques and PuraStat® hemostatic gel between January 2024 and April 2024. Data collected included patient demographics, bleeding characteristics, endoscopic findings, intervention details, and outcomes. Descriptive analysis was used to summarize the findings.

### 2.1 Ethical statement

The study was approved by the Indiana University Institutional Review Board (Protocol #23059). Informed consent for publication was obtained from the patient's parents or guardians.

## 3 RESULTS

### 3.1 Case 1

**Figure 1:**
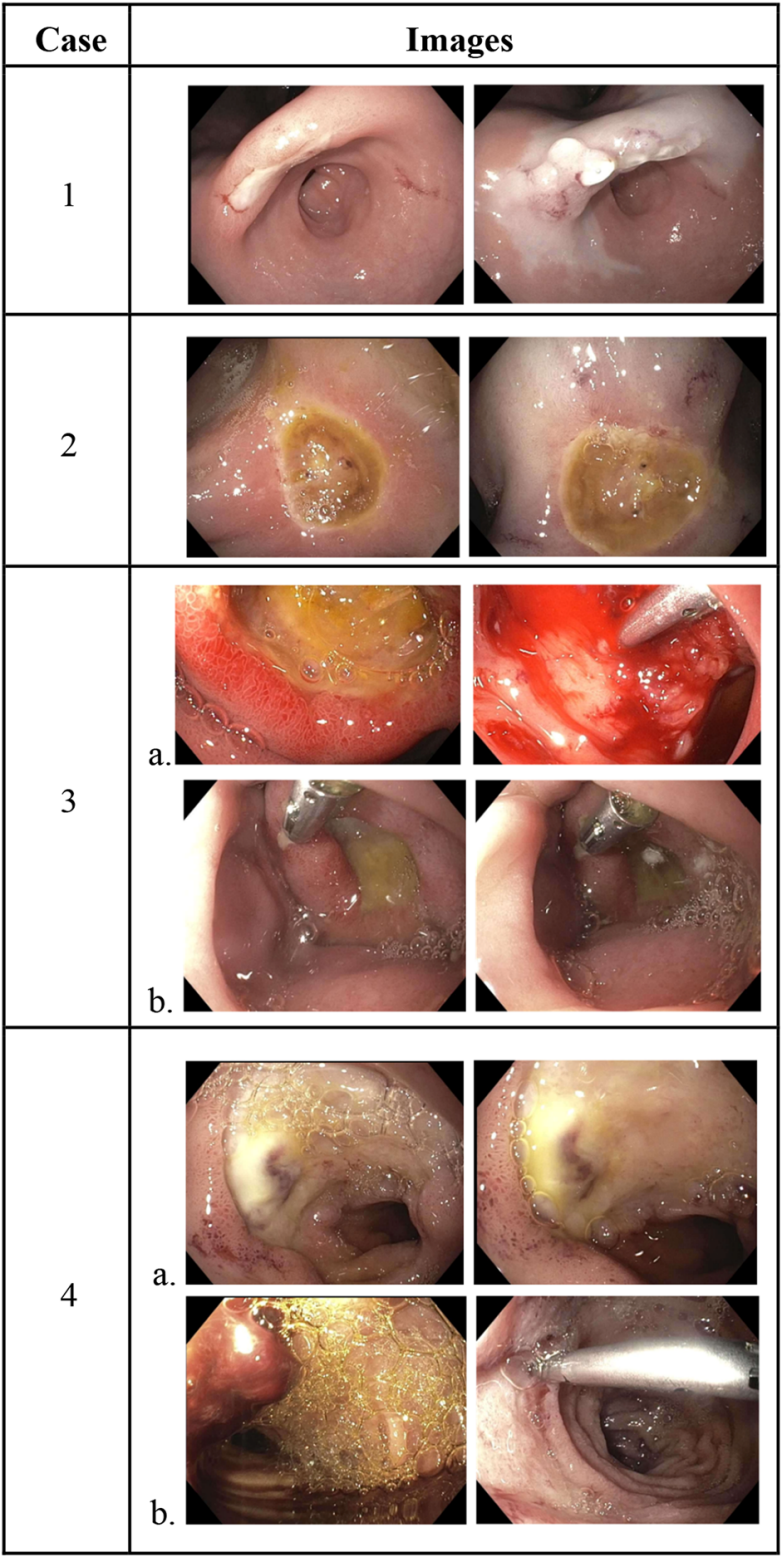
Endoscopic images and description of mucosal injury, location, and endoscopic interventions. 1. Incisura ulcer (IIc) before (left) and after (right) epinephrine injection and PuraStat. 2. Duodenal bulb ulcer (IIa) before (left) and after (right) epinephrine injection and PuraStat. 3a. Second portion of the duodenum ulcer (III) on initial endoscopy before (left) and after (right) PuraStat and clip placement. b. Second portion of the duodenum ulcer (III) on follow‐up endoscopy before (left) and after (right) PuraStat monotherapy. 4a. Duodenal bulb ulcer/site of previously contained perforation (IIa) before (left) and after (right) PuraStat monotherapy. b. Descending duodenal ulcer with oozing vessel after clot removal (Ib) shown with overlying clot (left) and after clip placement and PuraStat (right).

A 15‐month‐old male with tracheomalacia and feeding dysfunction underwent an EGD due to hematemesis, melena, tachycardia, and anemia with an acute drop in hemoglobin from 10.0 to 7.8 g/dL within 24 h, requiring a 15 mL/kg packed erythrocyte transfusion. High‐dose intravenous pantoprazole was initiated. The EGD revealed a nonbleeding gastric ulcer at the incisura with a flat pigmented spot (Forrest Classification IIc) (Figure 1, Image 1a). Epinephrine (0.1 mL of 0.1 mg/mL solution) was injected surrounding the ulcer base (Figure 1, Image 1b), then <1 mL of PuraStat® was applied to cover the ulcer (Figure 1, Image 1c). Postprocedural hemoglobin stabilized at 12.0 g/dL (from 10.7 g/dL), and no further hematemesis occurred. The cause of his ulcer remained unidentified. Biopsies revealed no evidence of gastritis or *Helicobacter pylori* organisms. Serum gastrin was normal. High‐dose oral pantoprazole was continued for 8 weeks before transitioning to a longer term once‐daily regimen. Follow‐up evaluation was unavailable at the time of publication.

### 3.2 Case 2

A 14‐year‐old female with cystic fibrosis, chronic pancreatitis post total pancreatectomy with islet cell autotransplantation, and type I diabetes mellitus presented with fatigue, abdominal pain, hematochezia, and anemia. Hemoglobin decreased from 12.6 to 7.9 g/dL over a period of 14 days and continued to decrease to 6.0 g/dL despite a 20 mL/kg packed erythrocyte transfusion upon hospitalization. History was notable for frequent use of nonsteroidal anti‐inflammatory drugs. High‐dose intravenous pantoprazole was initiated. A bleeding ulcer with a visible vessel (Forrest Classification IIa) was found on EGD at the gastro‐jejunal anastomosis (Figure 1, Image 2a). Epinephrine (0.5 mL of 0.1 mg/mL solution) was injected into all four quadrants surrounding the base of the ulcer, followed by PuraStat® application (<1 mL) (Figure 1, Image 2b). Repeat complete blood count testing approximately 14 h after the EGD showed a hemoglobin of 6.5 g/dL in the setting of three episodes of melena. She was given a 20 mL/kg erythrocyte transfusion, after which the melena subsided. It remained unclear whether the patient experienced ongoing bleeding after the procedure or if the anemia and brief episodes of melena were due to blood loss before and during the EGD. Repeat endoscopy was not performed as her hemoglobin remained stable with a value of 8.7 g/dL posttransfusion. High‐dose oral pantoprazole was continued for 12 weeks. Follow‐up hemoglobin was 12.9 g/dL 3 months later.

### 3.3 Case 3

A 15‐year‐old male with Crohn's disease, undergoing treatment with adalimumab and azathioprine, presented with abdominal pain and melena. Laboratory testing revealed a hemoglobin of 16.8 g/dL and a stool calprotectin of <5 μg/mg. Colonoscopy revealed no visible mucosal abnormalities. An EGD showed esophageal congestion and friability and a nonbleeding duodenal bulb ulcer (Forrest Classification III) (Figure 1, Image 3a). PuraStat® ( <1 mL) was applied, followed by hemostatic clip placement with minimal bleeding (Figure 1, Image 3b and 3c). There was no further hematochezia or melena noted during the admission. A follow‐up EGD was performed 3 days later for ulcer reassessment, showing the nonbleeding ulcer (Forrest Classification III) with a hemostatic clip in place (Figure 1, Image 4a). An additional 1 mL of PuraStat® was applied to the ulcer base as a preventative measure (Figure 1, Image 4b). Histopathology revealed eosinophilic esophagitis with 20–22 eosinophils per high power field in the esophagus, as well as acute inflammation in the stomach and duodenum. Abdominal pain resolved without additional melena before discharge home. He was asymptomatic at the time of follow‐up 4 weeks later and remained on high‐dose omeprazole for the treatment of eosinophilic esophagitis. Routine follow‐up endoscopy conducted 5 months later showed the duodenal ulcer and esophagitis had resolved.

### 3.4 Case 4

A 19‐year‐old male with a history of Duchenne muscular dystrophy developed melenic stools during an admission for cardiogenic shock and traumatic closed‐head injury. Laboratory testing revealed a hemoglobin of 4.6 g/dL, and computed tomography of the abdomen/pelvis showed descending duodenitis with a contained 2.9 cm duodenal perforation. He was treated with a transfusion of 1 unit of packed erythrocytes, bowel rest, and intravenous pantoprazole with improvement in melena. Resolution of the contained duodenal perforation was seen on fluoroscopic upper gastrointestinal imaging 7 days later. Feeding was resumed with interval redevelopment of melena and acute anemia, necessitating transfusion with 2 units of packed erythrocytes. At this time, an EGD was performed and revealed two areas of concern. One was a large duodenal bulb ulcer with a visible, nonbleeding vessel (Forrest Classification IIa) at the site of a recently contained perforation from 11 days earlier (Figure 1, Image 5a). It was unclear if this was the primary source of the UGIB, and to avoid disrupting the recent perforation site, PuraStat® (< 1 mL) was applied to this ulcer without any additional interventions (Figure 1, Image 5b). The other site was a medium‐sized ulcer with an adherent clot, found in the second portion of the duodenum (Figure 1, Image 6a). An oozing vessel was noted within the ulcer after clot removal (Forrest Classification Ib). A hemostatic clip was placed on the vessel followed by the application of PuraStat® gel (<1 mL) (Figure 1, Image 6b). The preoperative and postoperative hemoglobin levels were 7.7 and 6.9 g/dL, respectively. An additional unit of packed erythrocytes was given shortly after his procedure. No further melena was noted and the hemoglobin had increased to 10.0 g/dL about 1 month after discharge. Long‐term proton‐pump inhibitor therapy was continued.

## 4 DISCUSSION

This case series is the first to investigate the use of PuraStat® as adjunctive therapy or monotherapy for pediatric UGIB. Four patients, aged 1–19 years, had at least one ulcer identified as a source of active or recent bleeding. Three patients had duodenal ulcers, while one had a gastric ulcer. PuraStat® (0.5–1 mL) was used as an adjunct hemostatic method alongside hemostatic clip placement or epinephrine injections. In one patient, PuraStat® was used as monotherapy for a large duodenal ulcer at a recently contained perforation site 11 days prior. No complications or signs of continued bleeding requiring repeat endoscopy were noted. One patient experienced self‐resolving melena following intervention (Case 2), though it was unclear if this resulted from postprocedural bleeding or prior bleeding. Overall, in our small cohort, PuraStat® was easy to use and appeared to be beneficial in treating UGIB in children. No adverse events or technical failures were reported (Table 1). For optimal hemostasis, we found that applying a thin, even layer of the gel was most beneficial, as excessive application could result in detachment from the lesion due to the gel's weight, particularly in gravity‐dependent regions.

**Table 1:** Clinical characteristics of each case.

Case	Age (years)	Gender	Bleeding source	Forrest classification	Treatment modality	24‐h ΔHb (g/dL)	Rebleeding a. <7 day b. ≥7 day	Complications
1	1	Male	Ulcer— incisura of stomach	IIc	Epinephrine + PuraStat	↑ 1.3	a. No b. No	None
2	14	Female	Ulcer— duodenal bulb	IIa	Epinephrine + PuraStat	↓ 2.0	a. Yes^a^ b. No	None
3	15	Male	Ulcer— second portion of duodenum	III	a. Purastat + Clip b. Purastat	Not available	a. No b. No	None
4	19	Male	a. Ulcer— duodenal bulb b. Vessel within an ulcer— descending duodenum	a. IIa b. Ib	a. Purastat b. Clip + PuraStat	↓ 0.8	a. No b. No	None

Abbreviation: ΔHb, change in hemoglobin

^a^ Resolved without intervention.

The initial clinical use of PuraStat® in adults was primarily for postgastric tumor removal, with additional applications in cardiac and sinus surgery.^6^ Its efficacy and safety in adult gastrointestinal bleeding are well‐documented,^6^, ^7^, ^10^, ^11^ though little is known about its use in the pediatric population. Traditionally, metallic clips, epinephrine injections, and thermocoagulation techniques have been widely used for hemostasis in the setting of UGIB. Hemostatic powders were introduced within the last 15 years as an adjunct therapy for hemostasis. These powders function as a mechanical tamponade through activation of the coagulation cascade and increase of clotting factors, with literature reporting their efficacy and safety in pediatrics.^12^ Advantages of hemostatic powders include easy handling and immediate coverage of large surface areas, especially those in difficult anatomical regions. However, limitations include potential clogging of the catheter upon contact with moisture and obscuring the endoscopic view due to the opaque nature of the spray.^5^ In comparison, PuraStat® is a viscous, transparent hydrogel that does not clog within the application catheter nor obstruct the endoscopic view after use.

The financial implications of using PuraStat® are important to consider, especially when combined with other therapies. In the treatment of high‐risk ulcers, dual‐therapy is often employed to mitigate the risk of rebleeding. If PuraStat® is demonstrated to be as effective in pediatrics as in adults, it could be integrated as one of these dual‐therapy options. The cost of PuraStat® is comparable to that of hemostatic clips, though this can vary by institution and region. The ease of use as a clear gel could potentially lead to cost savings, though further economic evaluations are essential to assess its financial impact in pediatric UGIB management.

Several limitations should be considered. The small sample size limits the evaluation of PuraStat's® effectiveness and safety as a mono‐ or dual‐therapy agent. Most cases approached hemostasis via combination therapy, reducing the risk of re‐bleeding but increasing costs. The absence of routine follow‐up endoscopies prevented ulcer healing assessment. However, it is noteworthy that none of the patients experienced re‐bleeding requiring repeat endoscopy. Most ulcers were classified as Forrest II or III, with only one Forrest I lesion. Because of this, treatment encompassed both primary hemostasis for actively bleeding ulcers and preventive treatment for ulcers that had recently bled. Due to the limitation of Forrest I lesions, the potential in treating more severe cases may have been underestimated. The small size of our cohort limits the generalizability of our findings, emphasizing the need for large, prospective studies to validate our results.

Our experience with PuraStat® in a small cohort of children suggests that it may be a viable adjunctive option for managing UGIB in pediatric patients. Its rapid and effective hemostasis with ease of application is desirable for pediatric use. While PuraStat® appeared beneficial, conclusions about its safety and efficacy are limited by the small sample size and retrospective nature of this study. Future research is needed to compare PuraStat® with other adjunctive therapies and assess its effectiveness as monotherapy for pediatric UGIB.

## CONFLICT OF INTEREST STATEMENT

Brett J. Hoskins is a consultant for Mirum Pharmaceuticals, Inc. The remaining authors declare no conflicts of interest.
